# Smart Hydrogels for the Augmentation of Bone Regeneration by Endogenous Mesenchymal Progenitor Cell Recruitment

**DOI:** 10.1002/advs.201903395

**Published:** 2020-02-05

**Authors:** Philipp S. Lienemann, Queralt Vallmajo‐Martin, Panagiota Papageorgiou, Ulrich Blache, Stéphanie Metzger, Anna‐Sofia Kiveliö, Vincent Milleret, Ana Sala, Sylke Hoehnel, Aline Roch, Raphael Reuten, Manuel Koch, Olaia Naveiras, Franz E. Weber, Wilfried Weber, Matthias P. Lutolf, Martin Ehrbar

**Affiliations:** ^1^ Department of Obstetrics University Hospital Zurich University of Zurich Schmelzbergstr. 12 Zurich 8091 Switzerland; ^2^ Institute of Bioengineering School of Life Sciences and School of Engineering Ecole Polytechnique Fédérale de Lausanne (EPFL) Station 15 Lausanne 1015 Switzerland; ^3^ Institute for Dental Research and Oral Musculoskeletal Biology Center for Biochemistry University of Cologne Cologne 50931 Germany; ^4^ Department of Cranio‐Maxillofacial Surgery Oral Biotechnology and Bioengineering University Hospital Zurich Frauenklinikstrasse 24 Zurich 8091 Switzerland; ^5^ Faculty of Biology and BIOSS Centre for Biological Signalling Studies University of Freiburg Schänzlestr. 18 Freiburg 79104 Germany

**Keywords:** bone healing, bone morphogenetic proteins, growth factors, hydrogels, progenitor cells

## Abstract

The treatment of bone defects with recombinant bone morphogenetic protein‐2 (BMP‐2) requires high doses precluding broad clinical application. Here, a bioengineering approach is presented that strongly improves low‐dose BMP‐2‐based bone regeneration by mobilizing healing‐associated mesenchymal progenitor cells (MPCs). Smart synthetic hydrogels are used to trap and study endogenous MPCs trafficking to bone defects. Hydrogel‐trapped and prospectively isolated MPCs differentiate into multiple lineages in vitro and form bone in vivo. In vitro screenings reveal that platelet‐derived growth factor BB (PDGF‐BB) strongly recruits prospective MPCs making it a promising candidate for the engineering of hydrogels that enrich endogenous MPCs in vivo. However, PDGF‐BB inhibits BMP‐2‐mediated osteogenesis both in vitro and in vivo. In contrast, smart two‐way dynamic release hydrogels with fast‐release of PDGF‐BB and sustained delivery of BMP‐2 beneficially promote the healing of bone defects. Collectively, it is shown that modulating the dynamics of endogenous progenitor cells in vivo by smart synthetic hydrogels significantly improves bone healing and holds great potential for other advanced applications in regenerative medicine.

## Graphical abstract

Engineered biomaterials are used to trap and isolate bone progenitor cells during bone healing. Freshly isolated bone progenitor cells enable the identification of bioactive molecules that stimulate their recruitment. Identified recruitment‐stimulating molecules and bone morphogenetic protein are used to treat bone defects. Only the biomaterials‐controlled sequential, but not the simultaneous delivery of both factors improves bone healing.

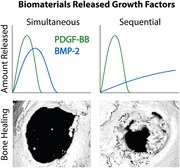

## 1 Introduction

Bone defects resulting from trauma or disease are currently treated by transplantation of autologous bone.^1^ However, due to limited supply and transplantation‐associated donor site morbidity, great efforts were put into development of off‐the‐shelf materials including recombinant osteogenic factors such as bone morphogenetic protein (BMP).^2^ Although BMP has shown promising results in preclinical evaluations, its application in clinical settings requires high doses that bear the risk of severe side effects.^3^ Hence, a significant effort has been directed toward the improved delivery of low‐dose BMP.^4^ A main reason for the low BMP‐2 efficacy can be attributed to the limited availability of endogenous mesenchymal progenitor cells (MPCs) at the site of large bone defects.^5^ Hence, it has been postulated that the augmentation of MPC recruitment by presentation of recruitment factors would promote BMP‐2‐mediated bone formation.[qv: 5a,d,6] Indeed, some growth factors such as stromal‐derived factor (SDF‐1), platelet‐derived growth factor (PDGF‐BB), fibroblast growth factor (FGF‐2), or vascular endothelial growth factor (VEGF) have been co‐delivered with BMP‐2.^7^ However, the simultaneous co‐delivery of these growth factors only moderately improved the healing of bone. Therefore, to closely recapitulate the inherent healing cascade, being first the mobilization and only later the differentiation of MPCs, sequential delivery strategies have been developed. Such staged dual growth factor release approaches relied on layer‐by‐layer assembly of coatings, core–shell microspheres, or heparin and fibronectin‐based affinities.[qv: 5d,8]

Despite the successful growth factor release from the aforementioned techniques, their complexity and limited tunability will likely restrict their widespread use for clinical applications. Optimally, synthetic fully defined extracellular matrices (ECMs) would be engineered to co‐deliver growth factors in a time‐controlled manner. We and others have previously developed synthetic poly(ethylene glycol) (PEG)‐based hydrogels, that serve as biocompatible and tunable cell niches.^9^ These fully defined niches allow the investigation of cell and growth factor functions in the absence of confounding and ill‐defined signals that are present in naturally derived ECM hydrogels. Furthermore, our PEG hydrogel that is cross‐linked by transglutaminase factor XIII (TG‐PEG) enables the modular assembly of building blocks and therefore the independent tailoring of parameters such as stiffness, proteolytic (e.g., matrix metalloproteinase (MMP)‐based) degradability, and sites for cell adhesion.^10^ Importantly, the flexible design of TG‐PEG hydrogels will concurrently enable the binding of multiple growth factors via different specific materials interactions and their tailored release.[qv: 10b,11] However, to the best of our knowledge, the sequential, controlled release of multiple factors from such rationally engineered, synthetic materials has not been achieved so far.

We hypothesized that for the unbiased in vitro screening of recruitment factors, freshly isolated MPC populations from healing bones should be employed. Therefore, we implanted our modularly engineered TG‐PEG hydrogels as an easy‐to‐harvest cell‐trap to isolate cells from bone defects. Cells were selected based on the stem cell antigen‐1 (Sca‐1), a marker found on multiple stem and progenitor cells,^12^ likely allowing the isolation of different healing‐relevant MPC populations. Thus, identified recruitment factors could then be ultimately used to design the next‐generation healing materials with MPC‐specific recruitment followed by differentiation, and thus enhance bone regeneration capacity.

The herein presented data show that healing bone defects contain a putative Sca‐1^+^ MPC population that spontaneously differentiates toward osteogenic lineages in vivo. In vitro assays showed that PDGF‐BB, FGF‐2, and epidermal growth factor (EGF) induce the mobilization, but at the same time inhibit the osteogenic differentiation of freshly isolated Sca‐1^+^ MPCs. Further, the most efficient migration factor, PDGF‐BB, significantly augmented the mobilization of endogenous Sca‐1^+^ cells in vivo. However, PDGF‐BB abrogated the bone healing activity of co‐delivered BMP‐2, indicating the need for a sequential delivery strategy. Importantly, engineered hydrogels with fast release of PDGF‐BB and the sustained delivery of low‐dose BMP‐2 promoted bone formation, and enhanced the effect of low‐dose BMP‐2. Collectively, our data show that the use of cell‐traps for the prospective isolation of tissue‐specific progenitor cells enables the study of individual healing responses in vitro and the consequent translation of the acquired knowledge to advanced cell‐free biomaterials‐based healing strategies.

## 2 Results

### 2.1 Optimizing Synthetic TG‐PEG Hydrogels for Bone Healing by Host Cell Recruitment

**Figure 1:**
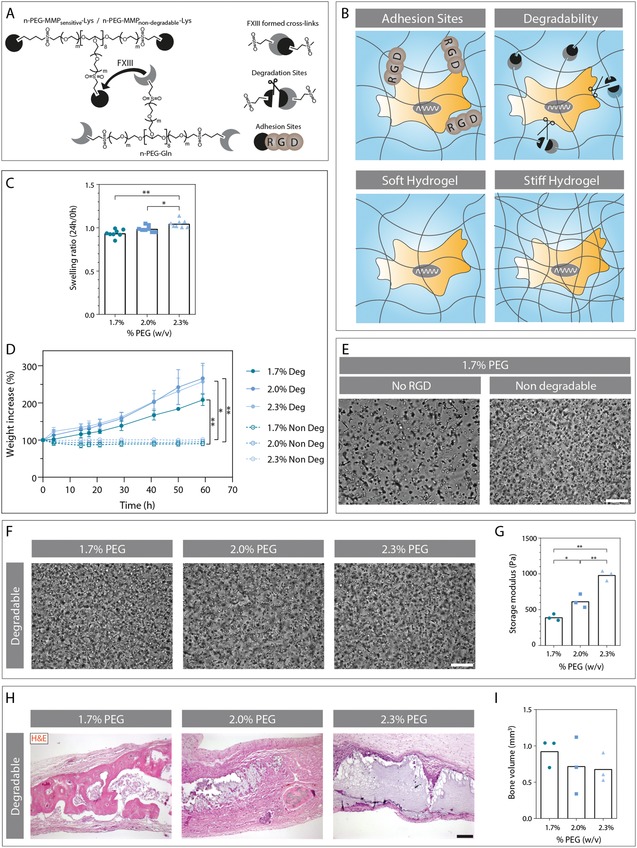
TG‐PEG hydrogels for bone healing. A) Scheme of modular‐designed FXIII cross‐linked TG‐PEG hydrogels. Modular building blocks comprise eight‐arm PEG precursors or cell‐adhesion peptide RGD, which are functionalized with either a glutamine (n‐PEG‐Gln) or a lysine (n‐PEG‐MMP_sensitive_‐Lys or n‐PEG‐MMP_nondegradable_‐Lys, and Lys‐RGD) comprising FXIII recognition sites. B) Schematic representation of hydrogel properties achieved by varying modular components. C) Swelling of TG‐PEG hydrogels formulated with different initial polymer concentrations. (*n* = 8). D) MMP‐1‐mediated degradation of different percentages TG‐PEG hydrogels containing MMP_sensitive_ (Deg) or MMP_nondegradable_ (Non Deg) cross‐links. Degradation of cross‐links results in the swelling of hydrogels (*n* = 5). E,F) Representative bright‐field images of hBM‐MPCs (2 × 10^6^ cells mL^−1^) encapsulated in TG‐PEG hydrogels after 5 days of 3D culture. E) When TG‐PEG hydrogels were formed in absence of RGD or by the use of non‐MMP degradable peptide linkers (scale bar = 200 µm). F) When TG‐PEG hydrogels containing 50 × 10^−3^ m RGD and MMP degradable peptide linkers were formed with increasing initial polymer concentration (scale bar = 200 µm). G) Corresponding storage moduli of the hydrogels at the indicated initial polymer concentration (*n* = 3). H,I) Healing of murine calvarial bone defects 4 weeks after implantation of hydrogels of different stiffness that contained 0.2 µg BMP‐2. H) H&E stainings (scale bar = 100 µm). I) Bone volume (BV) quantification by micro‐CT analysis (*n* = 3). Graphs show individual data points and means for independent hydrogels or defects. *p < 0.05, **p < 0.01 (one‐way ANOVA with Tukey–Kramer post hoc test).

In order to have a highly controlled and modular system to recruit and grow MPCs, the previously described TG‐PEG hydrogels were used.[qv: 10a,b] These synthetic TG‐PEG hydrogels are formed by the transglutaminase (TG) enzyme factor XIII (FXIII) that cross‐links equimolar blends of eight‐arm PEG precursors functionalized with either a peptide sequence containing a glutamine (n‐PEG‐Gln) or a lysine (n‐PEG‐MMP_sensitive_‐Lys or n‐PEG‐MMP_nondegradable_‐Lys) under physiological conditions. The use of MMP‐sensitive substrates and the cross‐linking in the presence of a lysine‐tagged RGD can be varied to turn TG‐PEG into a cell substrate to accurately match specific demands (**Figure** **1**A). As shown in earlier studies, the design of these TG‐PEG hydrogels allows their modular tailoring with respect to presentation of cell adhesion sites, proteolytic degradability, or mechanical properties (Figure 1B).[qv: 10c,11e,13] When formed at low initial polymer concentration (1.7–2.3% w/v), TG‐PEG hydrogels were stable and did not swell in a physiological buffer (Figure 1C). When exposed to MMP‐1, hydrogels formulated with MMP_sensitive_ cross‐links continuously swelled, indicating proteolytic degradation of the hydrogel, while hydrogels which contained only MMP_nondegradable_ cross‐links remained stable over the evaluated 60 h at 37 °C (Figure 1D). Cultures of human bone marrow‐derived MPCs (hBM‐MPCs) in soft TG‐PEG (1.7% w/v) hydrogels showed that in the absence of the integrin ligand RGD or the use of a nondegradable linker peptide (PEG‐MMP_nondegradable_‐Lys), cells could not efficiently spread (Figure 1E). This indicated the necessity of cell adhesion and degradable sites in the hydrogel backbones. Next, we investigated the effect of stiffness in encapsulated hBM‐MPCs in hydrogels ranging from 400 to 1000 Pa (Figure 1F,G). Even though, cell spreading was similar among these hydrogels, when hydrogels were implanted in calvarial defects after 4 weeks there was significantly more remodeling in the softest hydrogels (Figure 1H,I). Collectively, the softest hydrogels allowed the most efficient infiltration of healing‐associated cells, which likely contain a population of osteogenic MPCs.

### 2.2 Prospective Isolation of Healing‐Associated MPCs through Hydrogel Traps

**Figure 2:**
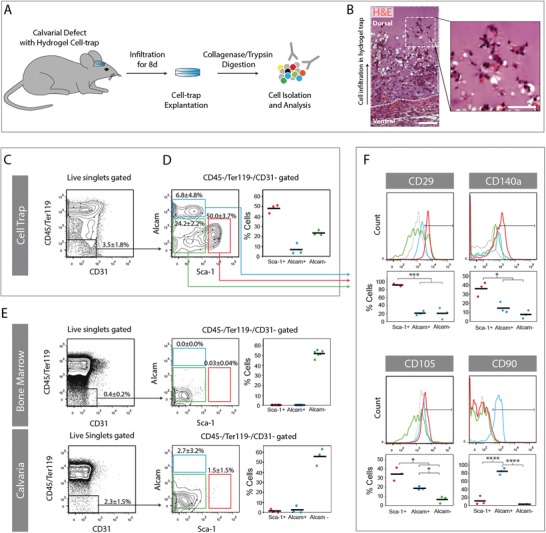
Sca‐1^+^ mesenchymal cells are enriched in the calvarial cell‐trap. A) Hydrogel cell‐traps were implanted for 8 days into cranial critical‐sized defects in mice. Infiltrated cells were harvested from cell‐traps by enzymatic digestion and further analyzed by flow cytometry. B) Histological section from the ventral side of an implanted hydrogel (H&E stain; scale bar left = 100 µm, scale bar right = 50 µm). Dotted line indicates the hydrogel, dashed square is a zoom‐in image. C–E) Graphs show a representative flow cytometric analysis of one independent experiment and the quantification of all independent experiments for implant entrapped cells (*n* = 3), bone marrow (*n* = 6), and crushed calvaria (*n* = 3). F) Phenotypic assessment of nonhematopoietic and nonendothelial Sca‐1^+^ (red), Alcam^+^/Sca‐1^−^ (blue), and Alcam^−^/Sca‐1^−^ (green) fractions (gray curve, isotype control). Graphs show individual data points and means for independent samples of implant entrapped cells (*n* = 3). *p < 0.05, ***p < 0.001, ****p < 0.0001 (one‐way ANOVA with Tukey–Kramer post hoc test).

To isolate MPCs that participate in bone regeneration, soft, proteolytically degradable, and RGD‐containing, but otherwise biologically inactive TG‐PEG hydrogels were placed in murine critical‐sized calvarial defects, where they served as a provisional healing matrix (**Figure** **2**A). We and others have shown that matrix mineralization occurs from 2 weeks post‐implantation,[qv: 11b,14] therefore in order to prevent stem cells from undergoing differentiation, implants were harvested at an earlier time point of 1 week post‐implantation. Remarkably, the implant material was infiltrated by various cell types 8 days post‐implantation (Figure 2B). Thus, we reasoned that this artificial niche could preserve the early cellular healing front, including endogenous and undifferentiated MPCs, and allow the facile isolation of these cells (thus, named from here on “cell‐trap”).

To show the participation of various stromal cell populations in the early healing events of calvarial bone, cell‐traps were harvested after 8 days of implantation and disintegrated by collagenase digestion. By fluorescence‐activated cell sorting (FACS), we could isolate hematopoietic, mesenchymal, and endothelial cell fractions. We observed a slightly higher (not significant) content of nonhematopoietic and nonendothelial cells (CD45^−^/Ter119^−^/CD31^−^; 3.5 ± 1.8%) in our cell‐traps as compared to freshly isolated bone marrow (0.4 ± 0.2%) and crushed calvaria (2.3 ± 1.5%) (Figure 2C–E). To further divide the mesenchymal cell fraction, we used Sca‐1 and Alcam, an earlier established marker pair for the isolation of endosteal niche cells from the bone marrow.^15^ Interestingly, cell‐traps contained large populations of Sca‐1^+^/Alcam^−^ cells (from here on termed Sca‐1^+^; 50.0 ± 3.7%; ≈30 000 cells per trap), as well as Alcam^+^/Sca‐1^−^ (from here on termed Alcam^+^; 6.8 ± 4.8%) and Alcam^−^/Sca‐1^−^ (from here on termed Alcam^−^; 24.2 ± 2.2%) cells (Figure 2D), corresponding to premature MPC subpopulations and osteoblasts, respectively.^15^ In contrast, bone marrow and calvarial bones contained significantly lower frequencies of Sca‐1^+^ cells (0.03 ± 0.04% and 1.5 ± 1.5%) and of Alcam^+^ cells (0% and 2.7 ± 3.2%), respectively (Figure 2E). While the frequency of Alcam^−^ cells was significantly higher in bone marrow and calvarial bones when compared to cell‐traps. This suggested that cells similar to those present at the endosteal niche of the bone marrow are significantly enriched in the healing bone defect.

CD105, CD140a, CD29, and CD90 have been identified as phenotypic markers of freshly isolated and cultured murine MPCs.[qv: 12d,14,16] Thus, we next analyzed the expression patterns of these markers within the isolated CD45^−^/Ter119^−^/CD31^−^ subpopulations (Figure 2F). A large fraction of Sca‐1^+^ cells was found to be positive for CD29 (91 ± 2.6%) and negative for CD90 (10.1 ± 9.0%). Alcam^+^ cells showed an opposite pattern regarding CD90 (83.8 ± 6.5%) and CD29 (20.3 ± 4.4%) expression, whereas Alcam^−^ cells showed a low expression of all tested markers. Within the Sca‐1^+^ population, CD140a and CD105 were expressed by a subpopulation of 36.4 ± 6.4% and 34.3 ± 5.8%, respectively. These data suggested that by the use of our cell‐traps we can isolate large numbers of healing‐contributing cells, which likely contain multiple distinct MPC subpopulations suitable to study healing processes.

### 2.3 Trapped Sca‐1^+^ Cells are Clonogenic and Differentiate into Different Mesenchymal Lineages

To further characterize the different trapped and freshly isolated cell populations, we next assessed their in vitro clonogenic and differentiation potential. CFU‐F assay conducted with the freshly isolated cell populations revealed a significantly (≈100‐fold) higher clonogenic frequency of the Sca‐1^+^ population (3.5 ± 1.3%) compared to Alcam^+^ (0.03 ± 0.01%) and Alcam^−^ (0.03 ± 0.02%) fractions, respectively (Figure S1A, Supporting Information). Sca‐1^+^ cells showed a spindle‐shaped morphology (Figure S1B, Supporting Information) and exhibited a strong proliferative potential. In contrast, cells from both Sca1^−^ fractions became senescent under evaluated culture conditions. Therefore, the clonogenic potential of the trapped CD45^−^/Ter119^−^/CD31^−^ fraction resided almost exclusively within the Sca‐1^+^ population. Furthermore, when cultured under commonly used differentiation conditions, Sca‐1^+^ cell differentiated into osteoblasts, adipocytes, and chondrocytes (Figure S1C, Supporting Information), demonstrating that these cells also fulfill the currently accepted in vitro criteria for mesenchymal stem/progenitor cells (MPCs).^17^

### 2.4 Sca‐1^+^ Cells Differentiate into Bone and Bone Marrow Cells

**Figure 3:**
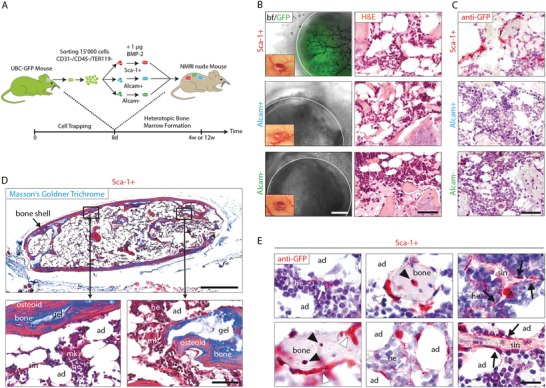
Trapped Sca‐1^+^ cells exhibit osteolineage potential in heterotopic bone marrow. A) Nonhematopoietic, nonendothelial Sca‐1^+^, Alcam^+^, and Alcam^−^ populations were harvested using cell‐traps from calvarial defects of mice ubiquitously expressing GFP (UBC‐GFP). 1.5 × 10^4^ freshly isolated cells of each fraction were incorporated into TG‐PEG gels (10 µL) supplemented with 1 µg BMP‐2 and implanted subcutaneously in immunocompromised NMRI nude receiver mice (*n* = 3). B,C) Heterotopic bone ossicles after 4 weeks of implantation. B) Macroscopic appearance (left column insert) as well as survival and participation of transplanted GFP^+^ cells in tissue formation (first column; fluorescent microscopy; scale bar = 500 µm) and H&E‐stained tissue sections (H&E second column; scale bar = 50 µm). C) Anti‐GFP stained tissue sections (GFP in red and counterstain with hematoxylin; scale bar = 50 µm). D,E) Tissue sections of heterotopic bone ossicles after 12 weeks of Sca‐1^+^ cell implantation. D) Masson's Goldner trichrome blue stain for detection of mineralized (bone) and nonmineralized (osteoid) areas (sin, sinusoids; mk, megakaryocytes; he, hematopoietic cell clusters; ad, adipocytes; gel, TG‐PEG hydrogel. Scale bar upper panel = 500 µm; Scale bar lower panels = 50 µm). E) Anti‐GFP stain (red, anti‐GFP; blue, hematoxylin counterstain; black triangle, osteocytes; white triangle, bone lining cells (osteoblasts); white arrow, reticular processes; black arrow, adventitial reticular cells of sinusoids. Scale bar = 20 µm.

To analyze their in vivo differentiation potential, we harvested cell‐traps from green fluorescent protein (GFP) mice (C57BL/6‐Tg(UBC‐GFP)30Scha/J). Fresh, prospectively isolated GFP^+^ and Sca‐1^+^, Alcam^+^, and Alcam^−^ cell populations were encapsulated in BMP‐2 releasing hydrogels and transplanted into subcutaneous pouches of immunocompromised nude recipient mice (HsdCpb:NMRI‐Foxn1) (**Figure** **3**A). 4 weeks post‐implantation, heterotopic ossicles comprising a laminar bone shell and a highly vascularized trabecular compartment were formed. Implants containing Sca‐1^+^ cells exhibited a strong fluorescent activity, indicating that Sca‐1^+^ cells successfully engrafted and expanded in vivo (Figure 3B). While in implants formed with Alcam^+^ or Alcam^‐^ cells, the GFP signal was almost absent. Immunostainings further confirmed that GFP^+^ host cells were only present in ossicles with Sca‐1^+^ cells. They further revealed that the cortical bone shell was exclusively formed by host‐derived cells and that transplanted Sca‐1^+^ cells localized in the trabecular compartment (Figure 3C).

To assess the fate of Sca‐1^+^ cells within the forming bone, ossicles were further analyzed 12 weeks after implantation when they appeared as complete bone marrows surrounded by a mineralized bone shell (Figure 3D). Histological analysis showed that within the newly formed heterotopic bone marrow, sinusoids, adipocytes, megakaryocytes as well as hematopoietic cell clusters existed. Immunohistochemical staining against GFP suggested that within the trabecular compartment, transplanted Sca‐1^+^ cells differentiated into osteoblasts, osteocytes, adipocytes, as well as adventitial reticular cells of sinusoids (Figure 3E). This indicates that Sca‐1^+^ cells contain populations that can undergo multiple differentiation paths within the forming bone and bone marrow.

### 2.5 Sca‐1^+^ Cells Spontaneously Differentiate into Osteocytes and Localize to Endosteal Regions

**Figure 4:**
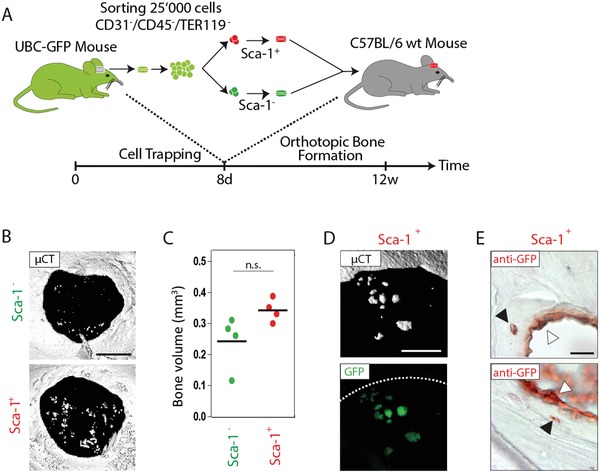
In healing bones, Sca‐1^+^ cells spontaneously form osteoblasts and osteocytes. A) 2.5 × 10^4^ fresh prospectively isolated, UBC‐GFP mice‐derived Sca‐1^+^ and Sca‐1^−^ cells were encapsulated in TG‐PEG hydrogels and implanted for 12 weeks into 4 mm calvarial bone defects in C57BL/6 receiver mice. B) Representative top views of 3D surface rendered micro‐CT reconstructions of treated defects (scale bar = 3 mm) and C) quantitative assessment of bone formation within defects, shown as individual as individual data points and means (*n* = 4, Student's *t*‐test, n.s. not significant). D) Contribution of Sca‐1^+^ cells to newly formed bone structures by sequential micro‐CT (upper panel) and fluorescence microscopy evaluation (lower panel; scale bar = 500 µm). E) Representative images of anti‐GFP stain‐based localization of GFP positive cells within the bone wound (black triangle, osteocytes; white triangle, bone lining cells (osteoblasts); scale bar = 20 µm).

Next, to assess their ability to participate in orthotopic bone regeneration, prospectively isolated Sca‐1^+^ or Sca‐1^−^ cells (comprising both Alcam^+^ and Alcam^−^ fractions) were encapsulated in hydrogels containing no exogenous BMP‐2, and implanted in calvarial bone defects (**Figure** **4**A). 12 weeks after implantation, bone defects treated with Sca‐1^+^ cells showed a trend toward an increased calcium deposition when compared to defects treated with Sca‐1^−^ cells (Figure 4B,C). A side by side comparison of 3D surface rendered micro‐computed tomography (micro‐CT) measurements with aligned fluorescence microscopy images revealed that GFP signals matched the calcified areas seen by micro‐CT analysis (Figure 4D). Furthermore, anti‐GFP immunohistochemical staining confirmed that Sca‐1^+^ cells accumulated in endosteal regions and localized within fully matured bone, indicating that they differentiated into osteoblasts and osteocytes, respectively (Figure 4E). Next, we wanted to confirm that Sca‐1^+^ cells spontaneously differentiate toward the osteogenic lineage and form bone also in ectopic sites. Indeed, when Sca‐1^+^ cells were hydrogel‐encapsulated in absence of growth factors and subcutaneously transplanted for 12 weeks, we found GFP expressing cell clusters seen in histological sections as bone nodules (Figure S2, Supporting Information). This suggests that the Sca‐1^+^ cell fraction contains one or more cell populations that can spontaneously differentiate toward the osteogenic lineage and might exert the same fate as in a bone defect. Collectively, these data indicate that Sca‐1^+^ cells isolated from cell‐traps are enriched in MPCs that can spontaneously differentiate to osteocytes and form new bone. Therefore, for the remainder of this manuscript freshly isolated, cell‐trap derived Sca‐1^+^ cell populations will be referred to as Sca‐1^+^ MPCs.

### 2.6 Growth Factors Promote a Differential In Vitro Migration of Sca‐1^+^ MPCs

**Figure 5:**
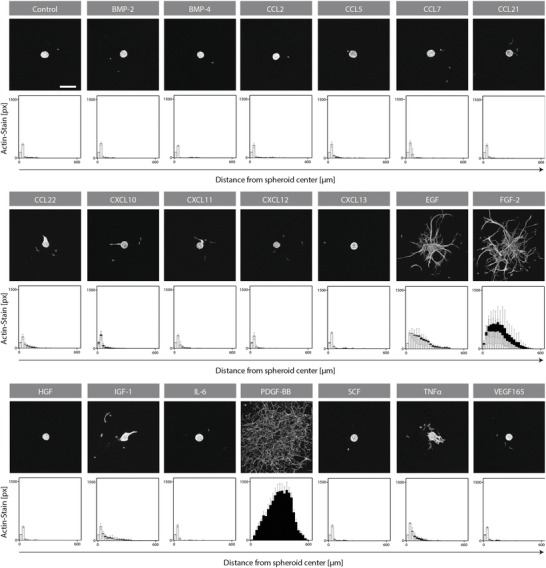
EGF, FGF‐2, and PDGF‐BB induce 3D migration of Sca‐1^+^ MPCs in an in vitro growth factor and cytokine screen. Spheroids consisting of 750 prospectively isolated Sca‐1^+^ MPCs were embedded in TG‐PEG hydrogels and cultured for 72 h in the presence of murine biomolecules: 10 ng mL^−1^ EGF (*n* = 5) and FGF‐2 (*n* = 4); 20 ng mL^−1^ IL‐6 (*n* = 5) and TNFα (*n* = 9); 30 ng mL^−1^ IGF^−1^ (*n* = 6); 50 ng mL^−1^ BMP‐2 (*n* = 5), BMP‐4 (*n* = 3), HGF (*n* = 4), and SCF (*n* = 5); 100 ng mL^−1^ PDGF‐BB (*n* = 5) and VEGF_165_ (*n* = 5); 150 ng mL^−1^ CCL2 (*n* = 7), CCL5 (*n* = 5), CCL7 (*n* = 6), CCL21 (*n* = 4), CCL22 (*n* = 6), CXCL10 (*n* = 11), CXCL11 (*n* = 5), CXCL12 (*n* = 10), and CXCL13 (*n* = 4). Collective cell migration (gray bars) and single‐cell migration (black) were quantified by automated image analysis. Data are depicted as mean ± SD. px, pixels. Scale bar = 200 µm.

To mimic their in vivo mobilization, aggregates of 750 freshly isolated Sca‐1^+^ MPCs were encapsulated within hydrogels and exposed to a library of 20 individual growth factors and cytokines with a previously described role in MPC migration. Under control culture conditions (i.e. no bioactive factor stimulation), time‐lapse microscopy did not reveal any migratory activity of Sca‐1^+^ MPCs. For treatment groups, the extent of radial outgrowth varied significantly, decreasing from PDGF‐BB and FGF‐2 to EGF and being negligible or even absent for all other factors (**Figure** **5**).

The observed effects with Sca‐1^+^ MPCs ranged from mesenchymal‐type movement for PDGF‐BB to collective migration of multicellular sprouts for FGF‐2 and EGF. Migration modalities are regulated by the differential expression of cell–cell or cell–ECM interaction (e.g. integrin and cadherin) molecules. During development and healing, this allows coordinated and oriented tissue guidance versus individual cell recruitment.^18^ Thus, our data suggest that the selected growth factors could increase the mobility and the recruitment of Sca‐1^+^ MPCs to the healing fracture by triggering different morphogenetic processes.

### 2.7 PDGF‐BB, EGF, and FGF‐2 Counteract Osteogenic Differentiation of MPCs

**Figure 6:**
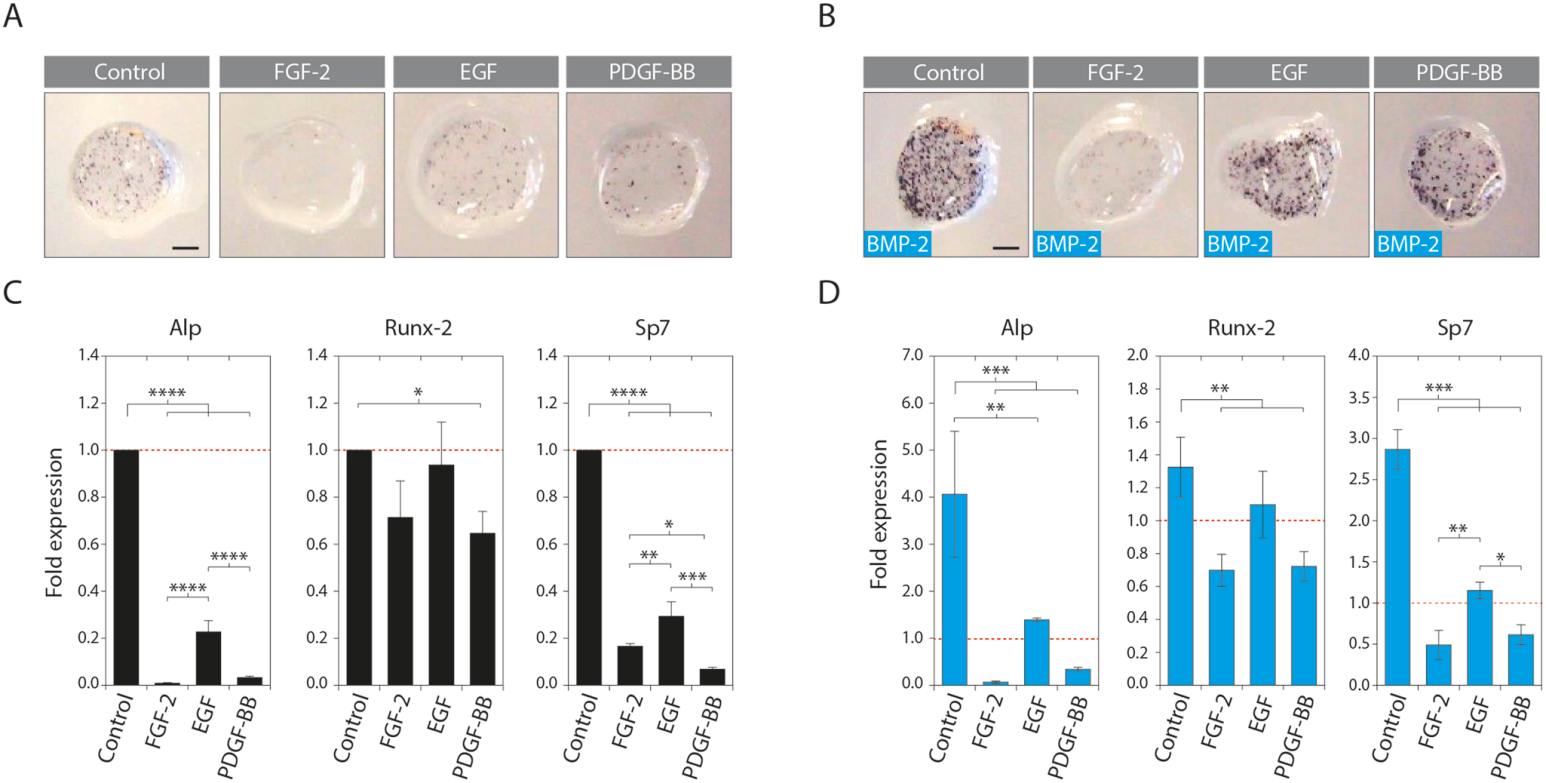
FGF‐2, EGF, and PDGF‐BB reduce BMP‐2‐induced osteogenic differentiation of calvaria‐derived MPCs in vitro. In vitro expanded mouse calvarial MPCs were encapsulated in TG‐PEG hydrogels at a concentration of 1.5 × 10^6^ mL^−1^ and cultured for 10 days in the presence of A,C) 0 and B,D) 100 ng mL^−1^ BMP‐2 as well as 50 ng mL^−1^ of the indicated growth factors (*n* = 3). A,B) Colorimetric assessment of alkaline phosphatase activity (scale bars = 1 mm). C,D) Gene expression analysis of early markers of osteogenesis. Data are depicted as mean ± SD for all panels, *p < 0.05, **p < 0.01, ***p < 0.001, or ****p < 0.0001 (one‐way ANOVA with Tukey–Kramer post hoc test).

During bone fracture healing, the morphogenetic processes migration, proliferation, and osteogenic differentiation occur sequentially. In fact, it has been shown that some migration and proliferation promoting biomolecules can counteract osteogenic differentiation.[qv: 8a,19] Therefore, to assess if delivery of PDGF‐BB, EGF, and FGF‐2 affects the spontaneous osteogenic differentiation of osteogenic progenitor cells, murine calvaria‐derived MPCs were encapsulated in biomimetic cell‐traps. The expression of early osteogenic differentiation markers was examined in the presence of these growth factors in vitro. PDGF‐BB, EGF, and FGF‐2 significantly lowered expression of alkaline phosphatase (ALP) and osterix (SP7), additionally PDGF‐BB reduced expression of Runx‐2 (**Figure** **6**A,C). Next, to mimic BMP‐mediated bone regeneration in vitro, hydrogel‐encapsulated MPCs additionally to treatment with the recruitment factors were exposed to 100 ng mL^−1^ BMP‐2. The presence of only BMP‐2 led to their efficient osteogenic differentiation as indicated by ALP activity (Figure 6B). However, treatment with 50 ng mL^−1^ PDGF‐BB, EGF, or FGF‐2 significantly reduced the BMP‐2‐induced ALP and osterix expression (Figure 6D). While the expression of Runx‐2 was also significantly reduced for PDGF‐BB and FGF‐2, only a mild (not significant) reduction was observed for EGF. To exclude that this observation is specific to murine MPCs, analogous experiments were performed with hBM‐MPCs (Figure S3, Supporting Information). Similarly, PDGF‐BB, and FGF‐2 significantly reduced the expression of ALP, Runx‐2, and osterix, while EGF only reduced the expression of ALP. Thus, these data suggest that tested doses of PDGF‐BB, EGF, and FGF‐2 are repressing in vitro osteogenic differentiation of MPCs likely in favor of their migration and proliferation.

### 2.8 FGF‐2, EGF, or PDGF‐BB‐Releasing TG‐PEG Hydrogels Do Not Induce Bone Healing

It is well accepted that stem cells upon exposure to specific microenvironments can differentiate toward terminally differentiated tissue cells.^20^ Thus, if osteogenic signals in healing bones are sufficiently strong, it should be expected that the accumulation of MPCs within a bone defect (by either recruitment or proliferation) can effectively stimulate bone healing. Therefore, we investigated if the selected migration factors FGF‐2, EGF, or PDGF‐BB as single treatment could improve the healing of calvarial defects (Figure S4, Supporting Information). Histological evaluations showed that all growth factor releasing hydrogels were completely remodeled, while control hydrogels remained intact. However, neither histological evaluations nor micro‐CT‐based analysis showed signs of bone regeneration in the growth factor releasing hydrogels. These observations indicate that FGF‐2, EGF, or PDGF‐BB could support the mobilization of healing‐associated MPCs, but at the same time osteogenic differentiation is not induced.

### 2.9 PDGF‐BB Promotes Perivascular MPC Recruitment

**Figure 7:**
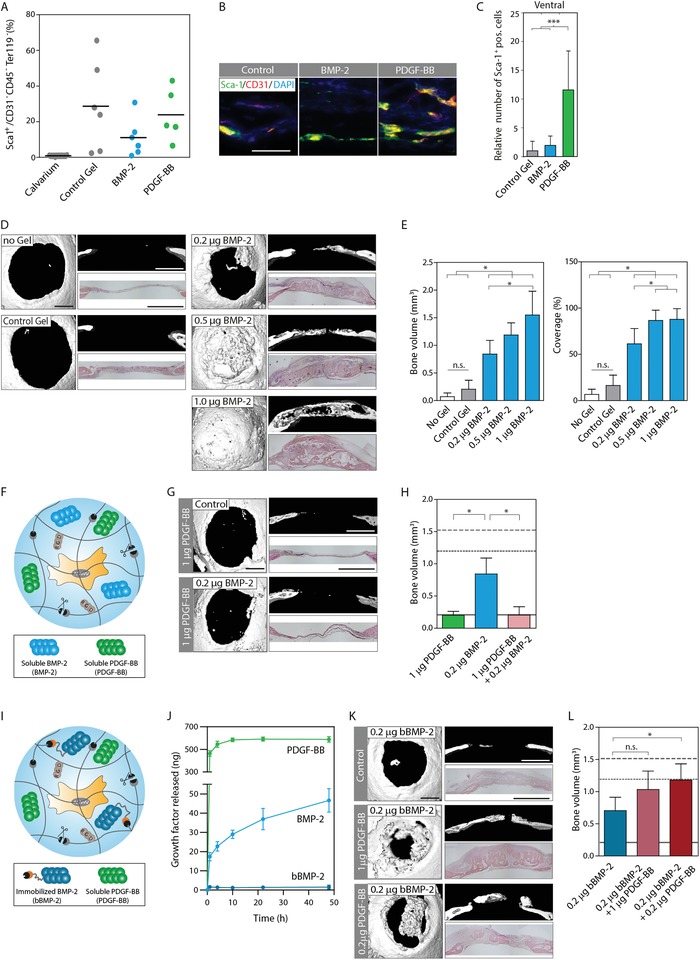
PDGF‐BB delivery enhances osteoinductive effect of BMP‐2 and increases recruitment of Sca‐1^+^ cells to bone defects. Bone defects ⌀ 4 mm were created in the left and right parietal bones of C57BL/6 mice. Defects were treated with preformed control hydrogel implants containing indicated amounts of soluble or matrix‐immobilized BMP‐2 and PDGF‐BB. Controls comprised defects that were left untreated or treated with empty hydrogel implants. A–C) The presence of MPCs in healing hydrogel implants was evaluated 8 days post‐craniotomy. A) Cells were released by collagenase and quantified by FACS. B) Tissue sections were stained against Sca‐1 and CD31 to localize Sca‐1^+^ cells and blood vessels. Representative immunofluorescence images of the most ventral side of the implant (scale bar = 50 µm). C) Quantitative assessment of Sca‐1^+^ immunohistology of defects. D–L) Healing bone defects 4 weeks post‐op. F) Schematic of strategy for delivery of soluble PDGF‐BB and soluble BMP‐2 or I) soluble PDGF‐BB and matrix‐immobilized BMP‐2. J) In vitro release of soluble and matrix‐immobilized growth factors by ELISA. D,G,K) Representative top (left panels) and side views (upper right panels) of 3D surface rendered micro‐CT measurements as well as H&E‐stained coronal cross‐sections (lower right panel) using D) soluble BMP‐2, G) soluble PDGF‐BB and soluble BMP‐2, or K) soluble PDGF‐BB and bound BMP‐2 (scale bar for all panels = 1 mm). E,H,L) Quantitative assessment of parameters for bone regeneration in response to different treatments, using E) soluble BMP‐2, H) soluble PDGF‐BB and soluble BMP‐2, or L) soluble PDGF‐BB and bound BMP‐2. BVs of control gels are indicated by a solid gray line, of 0.5 µg soluble BMP‐2 by a dotted line and of 1 µg soluble BMP‐2 by a dashed line. Data are depicted as mean ± SD for *n* = 6 independent defects. n.s. not significant, *p < 0.05, ***p < 0.001 (one‐way ANOVA with Tukey–Kramer post hoc test).

Next, to test if the best migration stimulating factor PDGF‐BB indeed augmented the number of Sca‐1^+^ MPCs at the site of treatment, calvarial bone defects were treated with empty, PDGF‐BB or BMP‐2‐releasing cell‐traps. After 8 days of implantation, the cell‐traps were harvested and processed for the analysis of recruited Sca‐1^+^ cells by FACS and immunological staining of histological sections. The overall cell composition of the healing bone analyzed by FACS showed no significant difference between treatment groups with respect to the recruitment of Sca‐1^+^ cells (**Figure** **7**A), endothelial cells (CD31^+^), or hematopoietic cells (CD45^+^, Ter119^+^; Figure S5, Supporting Information). In contrast, in histological evaluations, the ventral side of healing defects showed significantly higher numbers of Sca‐1^+^ cells after treatment with PDGF‐BB, but not with empty or BMP‐2 releasing hydrogels (Figure 7B,C). Furthermore, co‐stains against CD31 and Sca‐1 revealed the presence of blood vessels on the ventral side of the healing defect, together with Sca‐1^+^ cells which mostly localized in close proximity to these vessels (Figure 7B). This suggests that the native microenvironment of Sca‐1^+^ MPCs could be the perivascular niche, from where they could be mobilized and expanded during bone healing, a process that upon PDGF‐BB treatment might be mimicked and accelerated.

### 2.10 PDGF‐BB Abrogates BMP‐2‐Induced Bone Healing

Next, we reasoned that MPCs that were accumulated through PDGF‐BB delivery should efficiently undergo osteogenic differentiation and therefore low concentrations of BMP‐2 should result in efficient bone formation. To test BMP‐2 and PDGF‐BB co‐treatment, we had to first determine the dose‐dependent osteogenic activity of BMP‐2 in critical‐sized calvarial defects. Both histological and micro‐CT‐based evaluations of bone defects revealed fast transition from a BMP‐2 dose (0.2 µg) with a limited effect to a dose (1 µg) with almost complete bone healing (Figure 7D,E). While bone coverage was almost complete for 0.5 and 1.0 µg of BMP‐2, bone volume (BV) evaluations showed a nearly linear dose–response. Since treatment with 0.2 µg BMP‐2 led to only limited bone healing as compared to the control (Figure 7E), this condition was selected to further evaluate the combinatorial treatments. However, unexpectedly, hydrogels releasing combinations of 0.2 µg BMP‐2 and 1 µg PDGF‐BB completely abrogated the healing effects seen by hydrogels releasing only 0.2 µg BMP‐2 (Figure 7F–H). These findings together with our in vitro differentiation evaluations suggest that the presence of PDGF‐BB additionally to the stimulation of MPC recruitment inhibits their osteogenic differentiation.

### 2.11 Augmenting Bone Formation by Sequential Delivery of PDGF‐BB and BMP‐2

Next, we reasoned that concentration and timeframe of growth factors delivery must be aligned with the natural healing cascade of natural healing cascade of bone. Accordingly this growth factor delivery should first enhance the mobilization of MPCs and only later induce their osteogenic differentiation. Our in vitro release analysis showed that both PDGF‐BB and BMP‐2 are released in an initial burst. While the release of PDGF‐BB lasted only for 10 h, the release of BMP‐2 continued with a relative fast kinetic (Figure 7J). The growth factor payload is released through a combination of diffusion and hydrogel degradation, therefore, in the in vivo situation a faster release kinetic and therefore a faster exhaustion is expected. To obtain a persistent, low dose release of BMP‐2, which is in tune with the cell‐mediated degradation of the hydrogel, we have earlier established a streptavidin‐modified TG‐PEG (TG‐PEG‐Strep) hydrogel that efficiently binds biotinylated BMP‐2 (bBMP‐2; Figure 7I).[qv: 11c] We showed that this TG‐PEG‐Strep hydrogel‐bound bBMP‐2 can induce a localized osteogenic differentiation of human BM‐MPCs. Here, we confirmed the tight binding of bBMP‐2 to the TG‐PEG‐Strep hydrogel system (Figure 7J), while confirming that it is equally efficient in inducing the regeneration of bone as nonbound, fast‐release BMP‐2 (Figure 7E,L and Figure S6, Supporting Information).

Based on these in vitro investigations, the combined use of native PDGF‐BB and bBMP‐2 in streptavidin‐modified TG‐PEG hydrogels results in a sequential fast (diffusion‐based) release of PDGF‐BB and a continuous (hydrogel degradation‐coupled) slow release of bBMP‐2. Importantly, when we applied these smart two‐way dynamic release hydrogels containing a high concentration (1.0 µg) of fast‐release PDGF‐BB and a low concentration (0.2 µg) slow‐release bBMP‐2, we observed an improved bone healing compared to either bBMP‐2 or PDGF‐BB alone (Figure 7L). Furthermore, the use of even lower concentrations of PDGF‐BB (0.2 µg) for a two‐way dynamic release with bBMP‐2 (0.2 µg) resulted in an even further and significantly improved healing of bone when compared to bBMP‐2 alone. Together, these findings indicate that for improved bone healing, first the augmentation of MPC numbers at the site of bone regeneration, and subsequently their osteogenic differentiation needs to be controlled by balanced and staggered delivery of recruiting and differentiation factors. This is especially important for the design of next generation materials, which aim at co‐opting natural healing processes such as the diametrically opposed, and thus sequentially initiated mobilization and differentiation of stem and progenitor cells.

## 3 Discussion

In this study, we describe a new bioengineering approach for the screening of factors that promote bone healing by the recruitment and differentiation of endogenous MPCs. We show that the implementation of identified factors requires sophisticated biomaterials engineering to enable sequential growth factor release.

Fracture healing is accomplished by an incompletely characterized, complex interplay of various biomolecules and cells.[qv: 9a,21] While recently different mesenchymal stem and progenitor cell populations have been characterized, their exact functional relation and role in bone healing remain obscure.^22^ Additionally, it remains unknown how in vitro culture affects their function, which limits the information obtained through in vitro studies.^23^ Therefore, in this study we freshly isolated, bone regeneration‐derived mesenchymal (CD45^−^/Ter119^−^/CD31^−^) cells, which likely contain multiple distinct MPCs subpopulations, as a cell source to study and manipulate processes of bone regeneration.

Our provisional, fully defined TG‐PEG hydrogel cell‐traps were formed at a low stiffness, contained the integrin binding site RGD, and were degradable by MMP‐1. This optimized formulation of TG‐PEG hydrogels were infiltrated by a significant number of mesenchymal cells, allowing their prospective (here Sca‐1 and Alcam based) isolation of different subpopulations (Figure 2C,D). Sca‐1^+^‐based sorting of healing‐derived mesenchymal cells were significantly enriched for CFU‐Fs (Figure S1, Supporting Information), reaching a comparable frequency as described for hBM‐MPCs.[qv: 12a,14] The accumulation of Sca‐1^+^ MPCs (≈10^5^ Sca‐1^+^/Alcam^−^ cells per defect) with a high CFU‐F frequency at the wound site corresponds to the number of Sca‐1^+^ isolated from all femora and tibia of one mouse[qv: 12a] and is consistent with the earlier reported elevation of cell populations named skeletal stem cells (SSCs) and bone cartilage stromal progenitor cells in fractured versus uninjured bone.^24^

When subcutaneously transplanted in BMP‐2 containing PEG‐hydrogels, Sca‐1^+^ MPCs and their progeny contributed to the formation of ectopic ossicles, differentiated into trabecular osteocytes and osteoblasts, or localized to subsinosoidal structures of the heterotopic hematopoietic niches (Figure 3). In the absence of BMP‐2, Sca‐1^+^ MPCs formed isolated bone nodules and homed to the endosteum (Figure 4 and Figure S2, Supporting Information) comparable to CD146^+^ human MPCs.^25^ These data strongly suggest that trapped Sca‐1^+^ MPCs contain bone healing‐associated stem and progenitor populations similar to fracture callus‐derived SSCs^26^ and could be used to study novel bone healing approaches, including their mobilization to the site of regeneration.

In our 3D in vitro migration model, PDGF‐BB, EGF, and FGF triggered the mobilization of Sca‐1^+^ MPCs, while other known migration factors remained inactive (Figure 5). The inability of MPCs to respond to earlier identified mobilization factors can be explained by selective expansion of cellular entities or changes of cell function in cell culture expanded versus freshly isolated MPCs.[qv: 23a,27] Additionally, in contrast to Boyden chamber assays, in our 3D model the hydrogel‐encapsulated MPCs, comparable to their in situ mobilization, need to initiate the mesenchymal cell migration program, including cell polarization, cell adhesion, protease secretion, and actin strand contraction.^28^ Interestingly, all identified mobilization factors are released during physiological platelet activation and other early healing associated events,^29^ indicating their general role in the recruitment of MPCs in the native healing processes.

Our in vitro evaluations showed that the identified pro‐migratory factors PDGF‐BB, EGF, and FGF inhibited the osteogenic differentiation of murine (Figure 6) and human BM‐MPCs (Figure S3, Supporting Information), which is consistent with earlier observations.[qv: 8a] Additionally, treatment with individual recruitment factors did not improve calvarial bone healing (Figure S4, Supporting Information). These findings suggest that in our system, growth factor‐mobilized endogenous MPCs can promote bone healing only in the presence of an additional extrinsic osteogenic cue.

In PDGF‐BB‐treated calvarial defects, the total number of Sca‐1^+^ cells (by FACS analysis) did not change as compared to control and BMP‐2 treatment. This indicates that in healing bones not all Sca‐1^+^ cell populations respond to growth factor treatment (Figure 7A). Of note, PDGF‐BB‐releasing hydrogels promoted the mobilization of Sca‐1^+^ cells in the ventral part of the early (1 week) healing calvarial bone (Figure 7B,C). These Sca‐1^+^ cells mostly co‐localized with CD31^+^ endothelial cells, indicating their co‐infiltration with vascular structures. Since BMP‐2‐induced bone regeneration in PEG hydrogels occurs from the ventral side,[qv: 11b] this suggests that perivascular Sca‐1^+^ MPCs are crucial for bone regeneration.

Bone defects upon treatment with low‐dose BMP‐2 (0.2 µg per bone defect) healed (Figure 7D,E), while in co‐treatment with PDGF‐BB this healing was abrogated (Figure 7G,H). This finding together with the observed in vitro inhibition of MPC differentiation by PDGF‐BB suggests the need for a sequential release of first PDGF‐BB followed by BMP‐2. Our earlier described streptavidin‐modified hydrogels enabled diffusion‐controlled fast release of PDGF‐BB, and hydrogel remodeling‐controlled slow release of bBMP‐2.[qv: 11c,19] Using this sequential PDGF‐BB and bBMP‐2, release hydrogels (Figure 7I,J) significantly improved bone healing as compared to hydrogels that simultaneously released PDGF‐BB and bBMP‐2 or bBMP‐2 only (Figure 7K,L). These data together with our in vitro and in vivo evaluations of MPC mobilization suggest that PDGF‐BB leads to an early enrichment of MPCs at the defect site, while subsequent slow release of bBMP‐2 promotes their osteogenic differentiation. To our knowledge, this is the first report showing a significant improvement of bone regeneration by sequential over simultaneous growth factor delivery. Additionally, the fact that low‐dose (0.2 µg per defect) and high‐dose (1.0 µg per defect) PDGF‐BB were equally efficient in promoting bone healing by low‐dose BMP‐2 indicates that the concentrations of both factors must be tightly balanced and at no point in time be too high. Therefore, by carefully adjusting dose and release properties of PDGF‐BB and BMP‐2, bone healing could likely be further optimized.

Our study was restricted to recruitment and manipulation of MPCs involved in the healing of flat bone. It remains to be determined if the herein described principles also apply to healing of long bones. Additionally, earlier studies showed that in aged and diabetic animals the pool of MPCs is compromised.[qv: 5f,26,30] If and how in such compromised animals Sca‐1^+^ MPCs dynamics and healing with combined PDGF‐BB and BMP‐2 treatments are affected will need further evaluations. While in vitro release profiles have been evaluated (Figure 7J),[qv: 11c] in vivo release kinetics remain unknown. Also, at this stage a cell‐type selective infiltration of the implants cannot be excluded due to materials properties (including pore size, integrin adhesion, and degradability),^10^ which are significantly different from naturally occurring biomaterials.

The engineering of systems that specifically control biological processes by spatiotemporally tailoring the release of multiple factors will enable the study of healing mechanisms and the design of next‐generation smart healing materials. The herein used synthetic material allows the study of the natural healing cascade in absence of uncontrollable confounding signals present in naturally derived biomaterials. Moreover, by tailoring the cell‐trap degradation rate to the natural healing cascade, the spatial distribution of elements participating in bone healing can be conserved (Figure 2B). With respect to the here intended manipulation of the healing cascade, precisely balancing the migration, proliferation and differentiation of MPCs will be essential. To even further increase the body's healing capacity, the decoration of smart hydrogels with integrin binding sites that act synergistically with growth factor binding sites could be further explored.^31^ Finally, the correlation between the here reported murine MPCs and analogous human healing‐associated MPCs will be important for translational approaches.

## 4 Conclusion

Taken together, in this study we amplified the efficiency of BMP‐2 treatment by enhancing MPC recruitment to bone defects. To do so, we identified and manipulated cellular and molecular key players in bone healing. We envision the presented experimental sequence, which is based on i) prospective isolation of tissue stem and progenitor cells, ii) in vitro biomolecule screen, iii) rational design of a biomaterial for spatiotemporally controlled biomolecule delivery, and iv) in vivo efficacy assessment, to be applied in other areas of in situ tissue engineering, aiming at the augmentation of the natural healing cascade.

## 5 Experimental Section

##### Data Reporting

While experiments were not conducted in a randomized manner and by blinded investigators, all tissue specimens were processed and prepared for imaging by a blinded technician. Cell isolations from implants were conducted in three fully independent experimental rounds. Sample size of each experimental round included at least three animals per condition. For bone regeneration experiments, sample sizes of *n* = 5 were chosen based on effects to be 50% increased compared to controls and a standard deviation of 25%. The power was set at 0.8 and the acceptable error rate at 5%. Samples were excluded if the positioning of implanted materials was significantly shifted after surgical interventions. To exclude a time and surgery‐dependent bias, samples belonging to different experimental groups were conducted in a rotational basis and by a blinded investigator.

##### Preparation of TG‐PEG Hydrogels

As described previously,^10^ eight‐arm PEG precursors containing the pending factor XIIIa substrate peptides glutamine acceptor (n‐PEG‐Gln) or lysine donor with (n‐PEG‐MMP_sensitive_‐Lys) or without (n‐PEG‐MMP_nondegradable_‐Lys) an additional MMP‐sensitive linker were stoichiometrically mixed (final dry mass content as indicated—1.7%, 2.1%, or 2.3% w/v) in Tris‐Buffer (TBS, 50 × 10^−3^ m, pH 7.6) containing 50 × 10^−3^ m calcium chloride. Before use, all solutions were sterilized using a 0.22 µm syringe filter. When indicated, 50 × 10^−6^ m Lys‐RGD peptide (Ac‐FKGG‐RGDSPG‐NH_2_), indicated amounts of growth factors and/or cells were added to the precursor solution prior to initiation of cross‐linking by 10 U mL^−1^ thrombin‐activated factor XIIIa and vigorous mixing.

##### Equilibrium Swelling Measurements

Hydrogels were weighted after termination of FXIIIa cross‐linking and after 1 day of incubation in Tris‐Buffer (TBS, 50 × 10^−3^ m, pH 7.6). The swelling ratio was determined as the swollen gel mass divided by the gel's initial mass.

##### Degradation of Gels by MMP‐1

Hydrogels were immersed in 100 µL digestion buffer (50 × 10^−3^ m Tris, 50 × 10^−3^ m NaCl, 10 × 10^−3^ m CaCl_2_, 0.05% w/v Brij 35, pH 7.5) containing 50 × 10^−9^ m MMP‐1 (PeproTech, United Kingdom). The weight of hydrogels was repeatedly measured during incubation at 37 °C.

##### Rheological Analysis

Hydrogel stiffness was characterized by in situ rheometry. Gelation of 80 µL hydrogels at the indicated final dry mass content was analyzed on a rheometer (MCR 301, Anton Paar) equipped with 20 mm plate–plate geometry (PP20, Anton Paar) at 37 °C in a humidified atmosphere. The measuring gap size was set at 0.2 mm to ensure proper loading of the space between the plates and gel precursors. The evolution of storage modulus (*G*′) at a constant angular frequency of 1 Hz and constant shear strain of 4% was recorded for 30 min when equilibrium was reached.

##### Calvarial Defect Model

All in vivo experiments were approved by the veterinary offices of the canton of Zurich and were conducted in accordance with the Swiss law of animal protection. Adult wild‐type C57BL/6 mice (purchased from Harlan) or transgenic C57BL/6‐Tg(UBC‐GFP)30Scha/J female mice at the age of 10–12 weeks at the start of the experiments were used. 4 mm diameter craniotomies were created in the parietal bones of the skull, one on each side of, but not touching the sagittal suture. Defects that showed signs of dura injuries were not used for transplantations. Preformed sterile TG‐PEG hydrogel disks with a diameter of 4 mm were then placed in the defects. The TG‐PEG hydrogel disks were in advance produced by placing 14 µL of complete TG‐PEG solution supplemented with the indicated growth factors between two sterile hydrophobic glass microscopy slides (obtained by treatment with SigmaCote, Sigma‐Aldrich, cat. no. SL2) separated by spacers (≈1 mm thickness). After incubating the forming matrices at 37 °C for 30 min, hydrogel disks were released from the glass sides and stored in humidified atmosphere until their application. After placing the hydrogel into the defect, the skin was closed with sutures. Animals were euthanized 8 days post‐craniotomy for cell isolation and 4 or 12 weeks post‐craniotomy for analysis of bone healing.

##### Prospective Isolation and Flow Cytometry Analysis of Entrapped Cells

To isolate cells from defect sites, the defect sites were trimmed using surgical scissors in order to retrieve the hydrogel. Hydrogels were then fragmented by a scalpel and pieces were incubated in Dulbecco's modified Eagle medium (DMEM)/F‐12 + GlutaMAX (Gibco Life Technologies, cat. no. 31331‐028) supplemented with 1% v/v penicillin/streptomycin solution (P/S; Gibco Life Technologies, cat. no. 15140‐122) and 0.1% Collagenase A (Roche Applied Science, cat. no. 10103586001) at 37 °C for 50 min. After addition of trypsin (final conc. 0.025%; Gibco Life Technologies, cat. no. 25050) followed by a second incubation step for 10 min at 37 °C, cells were washed with FACS buffer (phosphate buffered saline (PBS) pH 7.2 with 1 × 10^−3^ m ethylenediaminetetraacetic acid) and filtered through a 100 µm cell strainer (BD Falcon, cat. no. 352350). Red blood cells were lysed by incubation with RBC lysis buffer (Biolegend, cat. no. 420301) for 10 min on ice and cells were washed and re‐suspended in FACS buffer. Cells were stained with monoclonal antibodies for 30 min at 4 °C. Employed antibodies can be found in the Supporting Information. Flow cytometric experiments and sorting were performed with a LSRII (BD Biosciences) and AriaIII (BD Biosciences), respectively. Gates were defined according to the fluorescence intensity of the isotype control. Data were analyzed using FlowJo 7.6.4. Software (TreeStar) and shown as contour plots or histograms of fluorescence intensity.

##### Cell Culture

Freshly sorted cells were cultured as adherent cultures on collagen‐coated plates at 37 °C with 5% CO_2_ in maintenance medium: Minimal essential medium alpha (MEMα; Gibco Life Technologies, cat. no. 22571‐020) with 10% v/v fetal calf serum (FCS; Gibco Life Technologies, cat. no. 10500) and 1% P/S supplemented with 5 ng mL^−1^ FGF‐2 (Peprotech, cat. no. 100‐18B).

##### Human Bone Marrow Cells

hBM‐MPCs were isolated from bone marrow aspirates of three healthy donors and cultured as described previously.^32^ Informed consent was obtained from the local ethical committee (University Hospital Basel; Prof. Dr. Kummer; approval date 26/03/2007 Ref Number 78/07). hBM‐MPCs were cultured at 37 °C in a humidified atmosphere and 5% CO_2_ using MEM α supplemented with 10% FCS, 1% P/S, and 5 ng mL^−1^ FGF^−2^.

##### CFU‐F Assay

10^2^ to 10^4^ isolated and freshly sorted cells were seeded on a 35 mm collagen‐coated dish (Techno Plastic Products, cat. no. 92006) and cultured for 11 days in maintenance medium supplemented with 5 ng mL^−1^ FGF‐2 at 37 °C with 5% CO_2_. After fixation with 4% paraformaldehyde for 10 min and stained with 0.05% w/v crystal violet solution for 30 min. Cell clusters counting > 50 cells were scored as colony.

##### In Vitro Differentiation

To assess in vitro differentiation, sorted Sca‐1^+^ cells were plated at 5 × 10^3^ cells well^−1^ in a collagen‐coated 96‐well plate (Techno Plastic Products, cat. no. 92096) in maintenance medium. The next day, medium was replaced with the following differentiation induction medium. To assess osteogenic differentiation, maintenance medium was replaced with osteogenic induction medium: DMEM high glucose (Gibco Life Technologies, cat. no. 41965‐039) with 10% v/v FCS and 1% v/v P/S modified with β‐Glycerophosphate (10 × 10^−3^ m; AppliChem, cat. no. A2253), dexamethasone (0.1 × 10^−6^ m; AppliChem, cat. no. A2153), and l‐ascorbic acid (0.05 mg mL^−1^; AppliChem, cat. no. A1052). Osteogenic induction medium was changed every 3–4 days. After 4 weeks, Alizarin red S staining was performed to assess for calcium deposition. To assess adipogenic differentiation, maintenance medium was replaced with adipogenic induction medium: DMEM low glucose (Gibco Life Technologies, cat. no. 31885‐02) with 20% v/v FCS and 1% v/v P/S modified with IBMX (0.5 × 10^−3^ m; AppliChem, cat. no. A0695), indomethacin (60 × 10^−6^ m; Sigma‐Aldrich, cat. no. I7378), and dexamethasone (1 × 10^−6^ m). Adipogenic induction medium was changed every 2–3 days. After 10 days, lipid droplet formation was measured using Oil red O staining. To assess chondrogenic differentiation, maintenance medium was replaced with chondrogenesis medium: DMEM high glucose with 10% v/v FCS and 1% v/v P/S modified with dexamethasone (0.1 × 10^−6^ m), l‐ascorbic acid (200 × 10^−6^ m), and TGF‐β3 (20 ng mL^−1^; PeptroTech, cat. no. 100‐36E). Chondrogenic induction medium was changed every 2–3 days. After 4 weeks, toluidine blue staining was performed to assess for chondrogenesis. In all experiments, sorted Sca‐1^+^ MPCs cultured in maintenance medium were used as controls.

##### Encapsulation of Single Cells and Microtissues in TG‐PEG Hydrogels

For encapsulating freshly isolated single dispersed cells or microtissues in TG‐PEG gels, cell or microtissue suspensions were diluted in maintenance medium without FGF‐2 and added to the complete TG‐PEG solution. Desired volumes of this mixture were then sandwiched between sterile hydrophobic glass microscopy slides (obtained by treatment with SigmaCote) separated by spacers (≈1 mm thickness) and clamped with binder clips. To prevent sedimentation of cells or microtissues, the forming matrices were slowly rotated at room temperature (RT) until the onset of gelation and then incubated for additional 30 min at 37 °C.

##### 3D Microtissue Migration Assay

For the following paragraph, maintenance medium without FGF‐2 was used. For microtissue formation, freshly harvested Sca‐1^+^‐enriched MPCs were suspended (final conc. 25 000 cells mL^−1^) in maintenance medium supplemented with 0.2% w/v methyl cellulose (Sigma‐Aldrich, cat. no. M0512). Droplets of 30 µL were placed in nonadhesive cell culture dishes (Greiner bio‐one, cat. no. 633180) and cultured overnight as hanging drops. The resulting spheroids (≈750 cells) were harvested in maintenance medium and washed once with maintenance medium. Sca‐1^+^ MPC microtissues were encapsulated in TG‐PEG hydrogels (final conc. ≈250 microtissues mL^−1^), hydrogels were thereafter released and transferred into a 24‐well plate (Techno Plastic Products, cat. no. 92024). Gels were then cultured for 4 days in 500 µL maintenance medium in the presence of the indicated murine biomolecules. All biomolecules were purchased as lyophilized powder from PeproTech and re‐suspended in 50 × 10^−3^ m Tris pH 7.6 supplemented with 0.1% bovine serum albumin (BSA, AppliChem, cat. no. A1391). Samples were fixed with 4% v/v paraformaldehyde in PBS, permeabilized with Triton X‐100 (Sigma‐Aldrich, cat. no. T‐8787) and stained for f‐actin with Rhodamine Phalloidin (Life technologies, cat. no. R415). Z‐stack images of microtissues were acquired at 10x magnification using a confocal laser scanning microscope (Leica TCS SP5). Cell migration was quantified by an automated image analysis script written in MATLAB (R2013a, MathWorks Inc., USA) that measures the distance of f‐actin stained pixels from the microtissue center and distinguishes between collective versus single cell migration. Pixels belonging to the connected network originating from the spheroid center were recognized as collective migration and residual pixels as single cell migration. The script's accuracy in distinguishing between collective and single cell migration was verified at multiple time points. Data were displayed as histogram depicting the amount of pixels relative to the migration distance.

##### Gene Expression Analysis Using qRT‐PCR

To measure the expression of osteogenic markers in TG‐PEG gels, mouse MPCs or human BM‐MPCs (*n* = 3) were encapsulated in TG‐PEG gels (1.7% PEG, 50 × 10^−6^ m RGD) at a final concentration of 1.5 × 106 mL^−1^. Gels were cultured for 10 days in MEMα, 10% FCS, 1% P/S, 10 × 10^−3^ m 4‐(2‐hydroxyethyl)‐1‐piperazineethanesulfonic acid, 1 × 10^−3^ m sodium pyruvate, 2 × 10^−3^ m L‐glutamine, 50 × 10^−6^ m, 10^−1^ L‐ascorbic acid, and 10 × 10^−3^ m β‐glycerol phosphate. The following recombinant growth factors were used for osteogenic differentiation experiments: human BMP‐2 (100 ng mL^−1^, produced as previously described^33^), FGF‐2, PDGF‐BB, EGF (each 50 ng mL^−1^, PeproTech, mouse or human, respectively). Medium and growth factors were replaced after 5 days. After 10 days, gels (40 µL) were digested in 50 µL collagenase A (2 mg mL^−1^, Roche) for 30 min at 37 °C. Next, cells were pelleted by a benchtop centrifuge (5415 R, Eppendorf) and total RNA was isolated from the cells by the RNeasy Micro Kit (Qiagen) according to the manufacturer's instructions. For quantitative real‐time PCR (qRT‐PCR), 100 ng RNA was converted into 20 µL cDNA by means of the high‐capacity cDNA reverse transcription kit (Applied Biosystems). qRT‐PCR was carried out using TaqMan Universal PCR Master Mix (Applied Biosystems) and the StepOnePlus Real‐Time PCR System (Applied Biosystems). The following TaqMan primer/probe sets were used for gene expression tests: Human: Hs01029144_m1 (ALPL); Hs01047973_m1 (RUNX2); Hs01866874_s1 (SP7). Mouse: Mm00475834_m1 (Alpl); Mm00501584_m1 (Runx2); Mm04209856_m1 (Sp7). Data were normalized on human Hs02758991_g1 (GAPDH) or mouse Mm99999915_g1 (Gapdh) and relative gene expression was calculated by the comparative Ct method.

##### Ectopic Bone Formation Model

For subcutaneous transplantation assays, 15 000 fresh (uncultured), prospectively isolated cells (from calvarial cell‐traps) were encapsulated in 10 µL TG‐PEG hydrogels in the presence or absence of total 1 µg BMP‐2. After completed polymerization, hydrogels were released from glass slides and stored in humidified atmosphere until application. Hydrogels were then implanted in subcutaneous pouches of immunocompromised nude mice (four samples per mouse; HsdCpb:NMRI‐Foxn1 mutant mice purchased from Harlan) at the age of 5–10 weeks and retrieved after 4 or 12 weeks post‐implantation.

##### Orthotopic Bone Formation Model

25 000 prospectively isolated, calvarial cell‐traps‐derived cells were encapsulated in 10 µL TG‐PEG hydrogels. After completed polymerization, hydrogels were released from glass slides and stored in humidified atmosphere until application. Hydrogels were then implanted in 4 mm diameter calvarial defects of 9–10 weeks old C57/BL6 mice (two samples per mouse) and retrieved 12 weeks post‐craniotomy.

##### Histological Staining and Immunohistochemistry of Ectopic and Orthotopic Bone Formation

Samples were fixed in 4% v/v formalin, decalcified with USEDECALC (MEDITE, cat. no. 40‐3310‐00) and embedded in paraffin. For histological stainings, sections (4 µm) were stained with hematoxylin & eosin (H&E) and Masson's trichrome (Sigma‐Aldrich, cat. no. HT15‐1KT). For anti‐GFP immunohistochemistry of ectopic bone formation, sections (4 µm) were stained with primary antibody against GFP (Abcam, cat. no. ab290). Alkaline phosphatase‐conjugated anti‐rabbit antibody was used as secondary antibody. Incubations were performed on Leica BondMax instruments using Refine HRP‐Kits (Leica DS9800) including all buffer‐solutions from Leica Microsystems, processed according to the manufacturers' guidelines. Antigen Retrieval was performed with Epitope‐Retrieval buffer 1 (Leica AR9961) at 100 °C for 10 min. Images of histological staining and immunohistochemistry were acquired using a Zeiss 200M inverted microscope. For orthotopic bone formation, calvaria were fixed with acetone for 4 h at −20 °C, decalcified in USEDECALC (MEDITE, D123501) and then embedded in cryomolds. Tissue sections of 5 µm thickness were cut on a cryostat (Leica CM3050S) and re‐fixed with acetone for 10 min. After rehydration in PBS for 10 min, soaking in blocking solution containing 1.5% BSA and 0.5% Tween‐20 they were exposed to anti‐GFP antibody (Abcam, cat. no. ab290) overnight at 4 °C. Then sections were incubated with a biotinylated secondary goat anti‐rabbit antibody for 1 h at RT, followed by incubation with an avidin‐biotin‐peroxidase complex for 1 h at RT (Vector Laboratories Inc., PK‐6101). Peroxidase was revealed by incubation with AEC (3‐amino‐9‐ethylcarbazole) red substrate (Vector Laboratories Inc., SK‐4200) prior blocking with 3% H_2_O_2_. Between individual incubation steps sections were rinsed three times with PBS containing 0.5% Tween‐20.

##### Microcomputed Tomography at Endpoints

After fixation in formalin and storage in PBS, complete skulls were scanned in a micro‐CT 40 (Scanco Medical AG) operated at energy of 70 kVp and intensity of 114 µA. Scans were executed at a high‐resolution mode resulting in a voxel size of 10 µm. In reconstructed images, bone tissue was segmented from background using a global threshold of 9.8% of maximum gray value. A cylindrical mask with a diameter of 3 mm was manually placed at the bone defect. BV, connectivity, and trabecular thickness within this mask were measured using the ImageJ plugin BoneJ.^34^ Coverage was measured in a dorsoventral projection of the cylindrical mask.

##### Recombinant Protein Production

Recombinant proteins BMP‐2 and TG‐Streptavidin were produced as described previously.[qv: 11c,33] BMP‐2 was biotinylated as described previously.[qv: 11c]

##### Immunohistochemistry and Histomorphometric Analysis of Cell Traps

Calvaria were harvested, fixed in 100% acetone for 4 h at −20 °C and embedded in cryomolds. Vertical sections of calvarial bones were cut on a cryostat (Leica CM3050S) at a thickness of 5 µm. The slides were re‐fixed with 100% acetone for 10 min and rehydrated in PBS for 10 min, soaked in blocking solution containing 1.5% BSA and 0.5% Tween‐20 in PBS for 45 min at RT and exposed to anti‐CD31 antibody (BD Pharmingen 550274) overnight in a humidified chamber at 4 °C. The slides were then incubated with a goat anti‐rat Alexa‐Fluor 546 secondary antibody for 1 h in the dark at RT. After washing, the slides were soaked in rat serum for 1 h at RT and exposed to anti‐Sca‐1 antibody (R&D Systems AF1226) for at least 2 h at RT. The slides were incubated with a donkey anti‐goat FITC‐conjugated secondary antibody for 1 h in the dark at RT. The specimens were washed three times in PBS, mounted in fluorescent mounting medium (DAKO S3023), and analyzed by fluorescent microscope (Leica BM550B). Images were processed in Fiji and Adobe Photoshop CS6 to create gray scale images. The relative number of Sca‐1^+^ cells on tissue sections was assessed by thresholding of gray scale images and quantification of pixels above the threshold (defined to exclude unspecific staining), and each value was normalized to the control gel).

##### Growth Factor Release In Vitro

TG‐PEG hydrogels (10 µL) containing 1 µg of PDGF‐BB, 0.2 µg of BMP‐2 or bBMP‐2 were incubated in 250 µL of 0.1% BSA in Tris buffer at 4 °C. After 1, 4, 10, 22, and 48 h, the solution was collected and frozen, and replaced by fresh buffer. Growth factor concentrations in these collected supernatants were then measured by the PDGF‐BB ELISA development kit (Peprotech, cat. no. 900‐K04) or BMP‐2 ELISA development kit (Peprotech, cat. no. 900‐T255) according to manufacturer's guidelines.

##### Statistical Analysis

Where sufficient samples (*n* > = 5) were available, normal distribution was assessed using a Kolmogorov–Smirnov test. All mean values were compared by one‐way analysis of variance (ANOVA) using MATLAB (R2013a, MathWorks Inc., USA) followed by a Tukey–Kramer post hoc test for pairwise comparison. Statistical significance was accepted for *p* < 0.05, and reported as follows *p < 0.05, **p < 0.01, ***p < 0.001, ****p < 0.0001. Further information is found in the particular figure legends.

## Conflict of Interest

The authors declare no conflict of interest.

## Supporting information

Supporting Information
