# Supplementary material for: Smart Hydrogels for the Augmentation of Bone Regeneration by Endogenous Mesenchymal Progenitor Cell Recruitment
Source: Adv Sci (Weinh). 2020 Feb 5;7(7):1903395. doi: 10.1002/advs.201903395 (PMC7141038; doi:10.1002/advs.201903395)
Supplement: Supplementary file 1 — Supporting Information [file ADVS-7-1903395-s001.pdf]

Copyright WILEY-VCH Verlag GmbH & Co. KGaA, 69469 Weinheim, Germany, 2020.

ADVANCED SCIENCE
Open Access

Supporting Information

for *Adv. Sci.,* DOI: 10.1002/advs.201903395

Smart Hydrogels for the Augmentation of Bone Regeneration by Endogenous Mesenchymal Progenitor Cell Recruitment

Philipp S. Lienemann, Queralt Vallmajo-Martin, Panagiota Papageorgiou, Ulrich Blache, Stéphanie Metzger, Anna-Sofia Kiveliö, Vincent Milleret, Ana Sala, Sylke Hoehnel, Aline Roch, Raphael Reuten, Manuel Koch, Olaia Naveiras, Franz E. Weber, Wilfried Weber, Matthias P. Lutolf, and Martin Ehrbar**

Copyright WILEY-VCH Verlag GmbH & Co. KGaA, 69469 Weinheim, Germany, 2018.

Supporting Information

Smart hydrogels for the augmentation of bone regeneration by endogenous mesenchymal progenitor cell recruitment

Philipp S. Lienemann, Queralt Vallmajo-Martin, Panagiota Papageorgiou, Ulrich Blache, Stéphanie Metzger, Anna-Sofia Kiveliö, Vincent Milleret, Ana Sala, Sylke Hoehnel, Aline Roch, Raphael Reuten, Manuel Koch, Olaia Naveiras, Franz E. Weber, Wilfried Weber, Matthias P. Lutolf and Martin Ehrbar**

FACS Antibodies: For flow cytometry analysis, the following antibodies were used: Sca-1-PE-Cy5 (D7; cat. no. 108109), Alcam-PE (eBioALC48; eBioscience, cat. no. 12-1661), CD31-PerCP (390; eBioscience, cat. no. 46-0311-82), Ter119-APC-Cy7, CD45-APC-Cy7 (30-F11; cat. no. 103115), CD140a-APC (APA5; cat. no. 135907), CD90.2-PE-Cy7 (53-2.1; cat. no. 140309), CD29-Pacific blue (HMβ1-1; cat. no. 102224) and CD105-Alexa 488 (MJ7/18; cat. no. 120405). For cell sorting, the following antibodies were used: Sca-1-PerCP (D7; cat. no. 108121), Alcam-PE (eBioALC48; eBioscience, cat. no. 12-1661), CD31-Pacific blue (390; cat. no. 102421), Ter119-APC-Cy7 (TER-119; cat. no. 116223) and CD45-APC-Cy7 (30-F11; cat. no. 103115). Sytox Blue dead cell stain (Invitrogen, cat. no. S34857) was used to evaluate cell viability and compensation was performed using the AbC™ anti-Rat⁄Hamster Bead Kit (Life technologies, cat. no. A10389).

List of abbreviations

AEC	3-amino-9-ethylcarbazole
ALP	Alkaline phosphatase
bBMP-2	Biotinylated BMP-2
BCSP	Bone cartilage stromal progenitor
BMP-2	Bone morphogenetic protein-2

BSA	Bovine serum albumin
BV	Bone volume
CFU-F	Fibroblast colony-forming unit
ECM	Extracellular matrix
EGF	Epidermal growth factor
FACS	Fluorescence-activated cell sorting
FCS	Fetal calf serum
FGF-2	Fibroblast growth factor-2
GFP	Green fluorescent protein
Gln	Glutamine
H&E	Hematoxylin & eosin
hBM-MPC	Human bone marrow-derived mesenchymal progenitor cell
Lys	Lysine
MEMα	Minimal essential medium alpha
Micro-CT	Micro-computed tomography
MMP	Matrix metalloprotease
MPC	Mesenchymal progenitor cell
n.s.	Not significant
P/S	Penicillin/streptomycin solution
PBS	Phosphate-buffered saline
PDGF-BB	Platelet-derived growth factor BB
PEG	Poly(ethylene glycol)
Px	Pixels
qRT-PCR	Quantitative real-time polymerase chain reaction
RBC	Red blood cell
RT	Room temperature
Sca-1	Stem cells antigen-1
SD	Standard deviation
SP7	Osterix
SSC	Skeletal stem cell
TBS	Tris-Buffer
TG-PEG	Transglutaminase factor XIII PEG
UBC-GFP	Mice ubiquitously expressing GFP
WT	Wild type

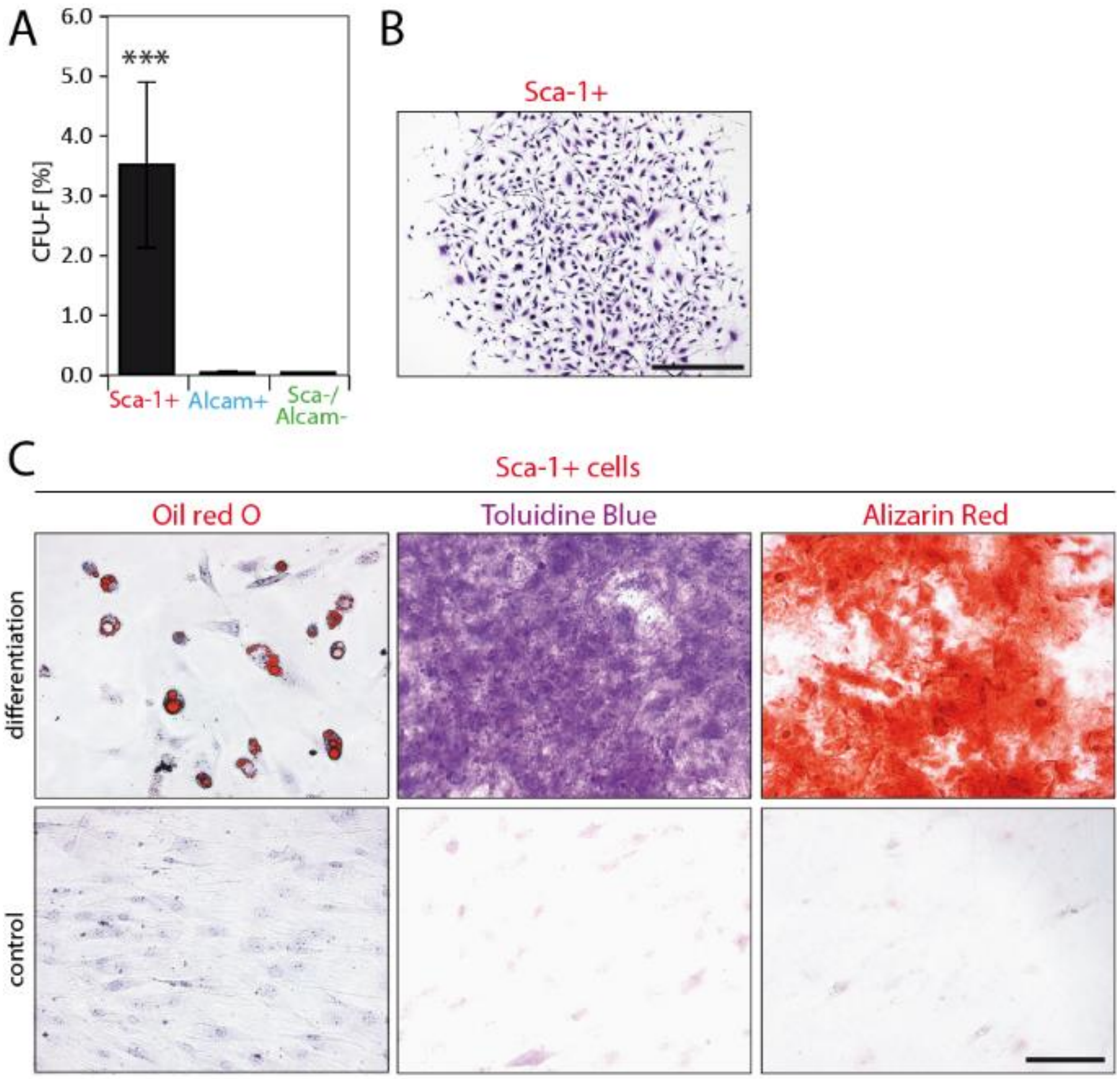

Figure S1. Trapped Sca-1$^+$ cells show mesenchymal progenitor potential *in vitro*. Non-hematopoietic and non-endothelial cells from a calvarial cell-trap were sorted into Sca-1$^+$, Alcam$^+$ and Sca-1$^-$/Alcam$^-$ cell populations. (**A**) To assess their clonogenic potential $10^2 – 10^4$ freshly isolated Sca-1$^+$, Alcam$^+$ or Sca-1$^-$/Alcam$^-$ cells were cultured for 11 days on a 35 mm dish then colonies counted (colony > 50 cells; n = 6). (**B**) Representative image of a Sca-1$^+$ colony after 11 days (crystal violet stain; scale bar = 1000 µm). (**C**) *In vitro* differentiation potential of Sca-1$^+$ cells was examined by incubation with differentiation medium for 10 days for adipogenesis (Oil red O stain) as well as for 28 days for chondrogenesis (toluidine blue stain) and for osteogenesis (alizarin red stain) (upper panel). As a control Sca-1$^+$ cells were expanded in standard culture medium (lower panel). (n = 3; scale bar = 200 µm). Data is depicted as mean ± SD. *** $p < 0.001$ (1-way ANOVA with Tukey-Kramer *post hoc* test).

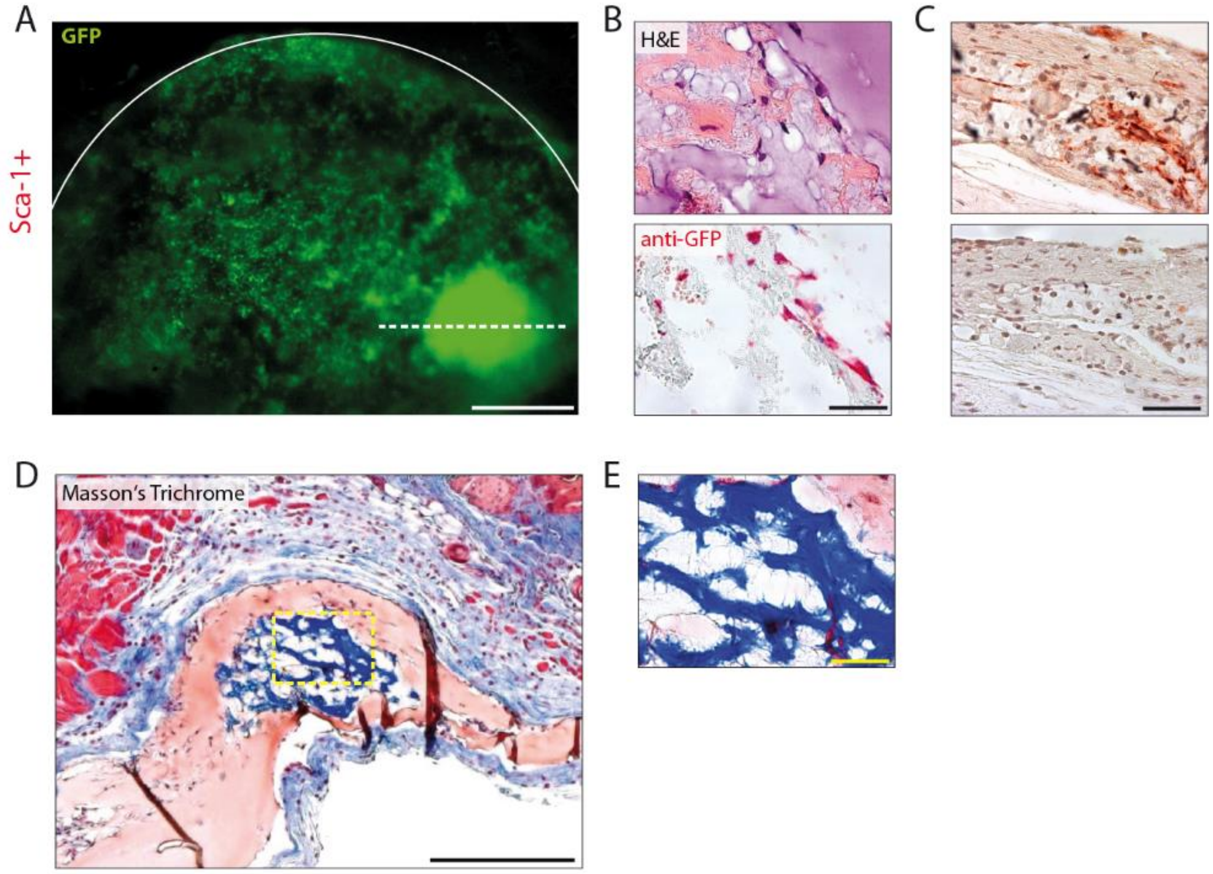

Figure S2. Trapped Sca-1$^+$ cells exert osteolineage potential in highly condensed areas. Cell-traps were implanted into 5 mm calvarial defects in mice ubiquitously expressing GFP (UBC-GFP) and cells were harvested after 8 days. Then, cells were sorted and 1.5 x 10^4 Sca-1$^+$ MPCs were encapsulated in TG-PEG gels (10 µl) in absence of BMP-2 and implanted into a subcutaneous pouch in the back of an immunocompromised receiver mouse. **A)** Fluorescent microscopy image showing the implant 12 weeks after implantation (scale bar = 500 µm). Histological sections through the position of the white dotted line were stained with (**B**) hematoxylin and eosin (H&E, top) or anti-GFP (red, bottom, scale bar = 40 µm), or with (**D and E**) Masson's Goldner trichrome staining (**D**) at low magnification (scale bar = 250 µm) and (**E**) at high magnification (scale bar = 40 µm). (**C**) Implant area stained with GFP-specific antibody (top) and IgG control (bottom, scale bar = 40 µm).

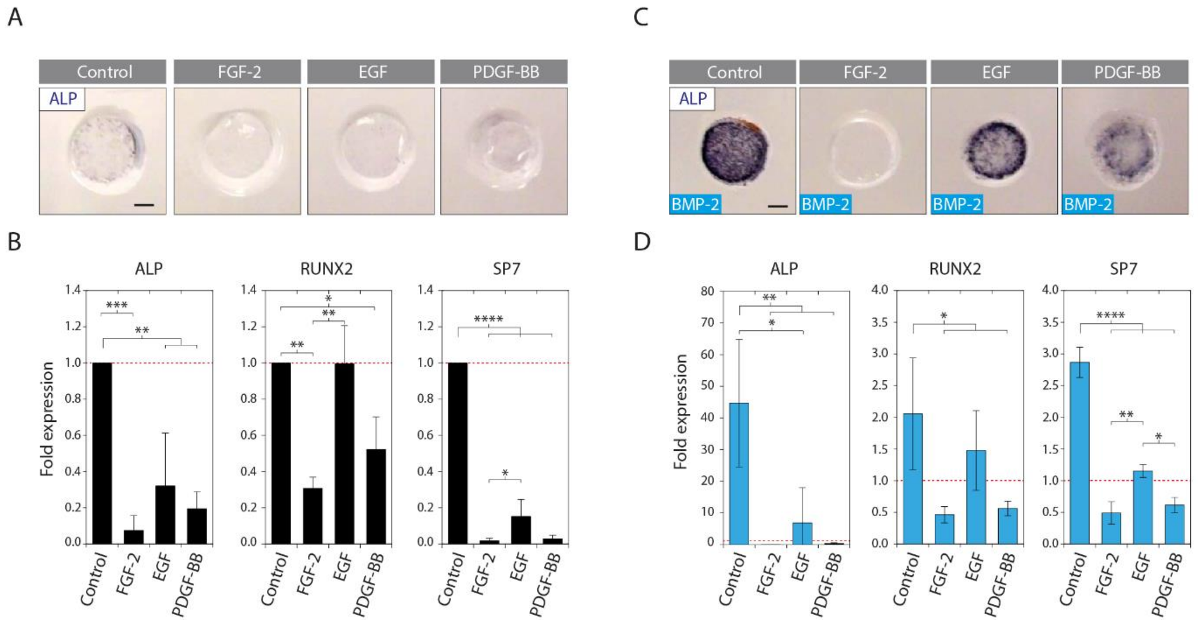

Figure S3. FGF-2, EGF and PDGF-BB reduce BMP-2-induced osteogenic differentiation of human bone marrow-derived MPCs (hBM-MPC) *in vitro*. hBM-MPCs were encapsulated in TG-PEG hydrogels at a concentration of 1.5 x 10^6 ml^{-1} and cultured for 10 days in presence of (**A and B**) 0 and (**C and D**) 100 ng ml^{-1} BMP-2 as well as 50 ng ml^{-1} of the indicated growth factors (n = 3). (**A and C**) Colorimetric assessment of alkaline phosphatase activity. Scale bars = 1 mm. (**B and D**) Gene expression analysis of early markers of osteogenesis. Data is depicted as mean ± SD for all panels. * $p < 0.05$, ** $p < 0.01$ *** $p < 0.001$, **** $p < 0.0001$ (1-way ANOVA with Tukey-Kramer *post hoc* test).

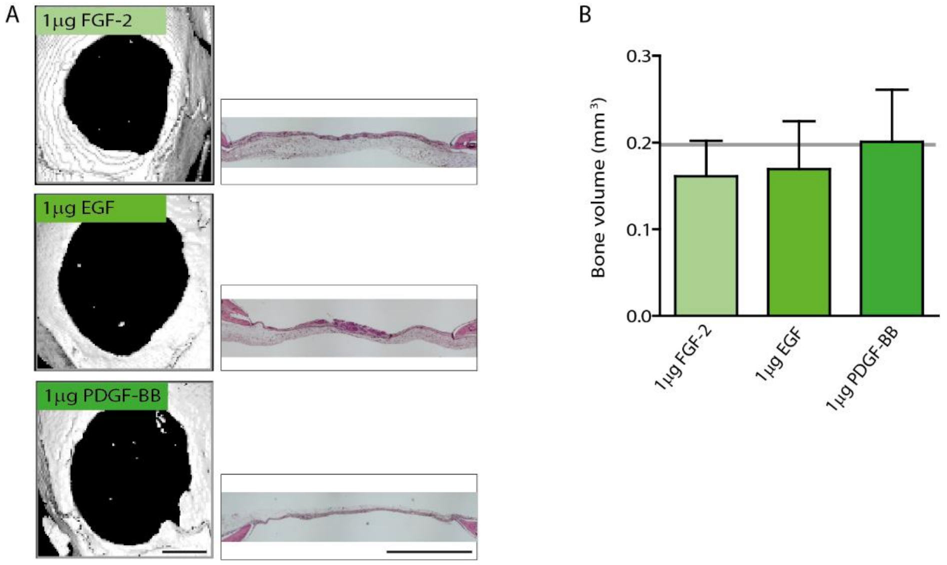

Figure S4. Single Factors FGF-2, EGF and PDGF-BB do not support the healing of bone defects. Preformed hydrogel implants containing indicated amounts of soluble FGF-2, EGF and PDGF-BB were placed in ⌀ 4 mm bone defects created in the left and right parietal bones of C57BL/6 mice. Healing of bone defects was allowed to take place for 4 weeks post-op. (**A**) Representative top (left panels) of 3D surface rendered micro-CT measurements as well as H&E stained coronal cross sections (right panels) using soluble FGF-2, EGF and PDGF-BB (scale bars = 1 mm). (**B**) Quantitative assessment of bone volume in response to different

treatments. The gray line indicates the mean bone density of treatments with control hydrogels. Data is depicted as mean ± SD for n = 6 independent defects (1-way ANOVA with Tukey-Kramer *post hoc* test).

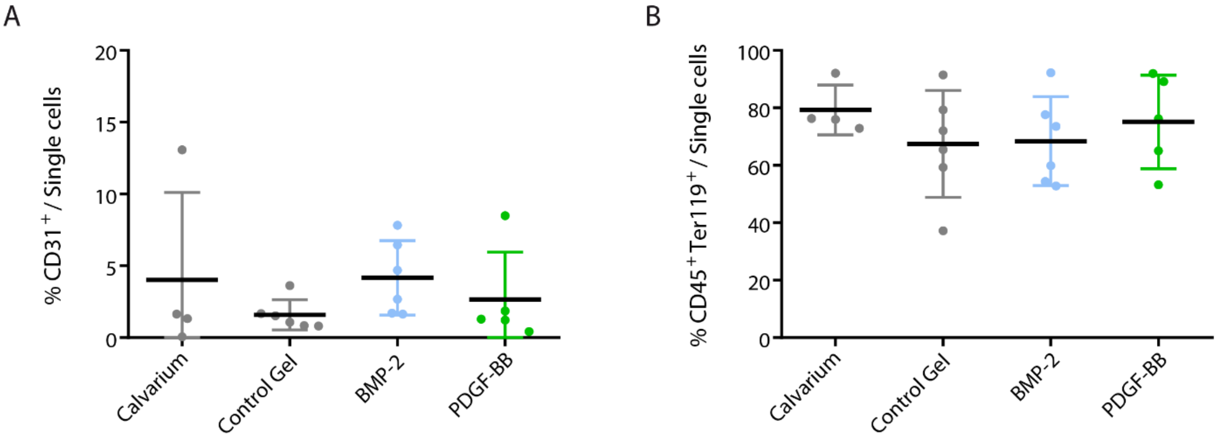

Figure S5. Recruitment of endothelial and hematopoietic cells to healing bone defects. Bone defects ⌀ 4 mm were created in the left and right parietal bones of C57BL/6 mice. Defects were treated with preformed hydrogel implants containing BMP-2 or PDGF-BB. Controls comprised defects that were left untreated or treated with empty hydrogel implants. 8 days post-craniotomy the healing hydrogel implants were digested by collagenase, and the presence of (**A**) endothelial cells (CD31$^+$) and (**B**) hematopoietic cells (CD45$^+$ Ter119$^+$) was quantified by FACS. Data is depicted as mean ± SD for n ≥ 4 independent defects (1-way ANOVA with Tukey-Kramer *post hoc* test).

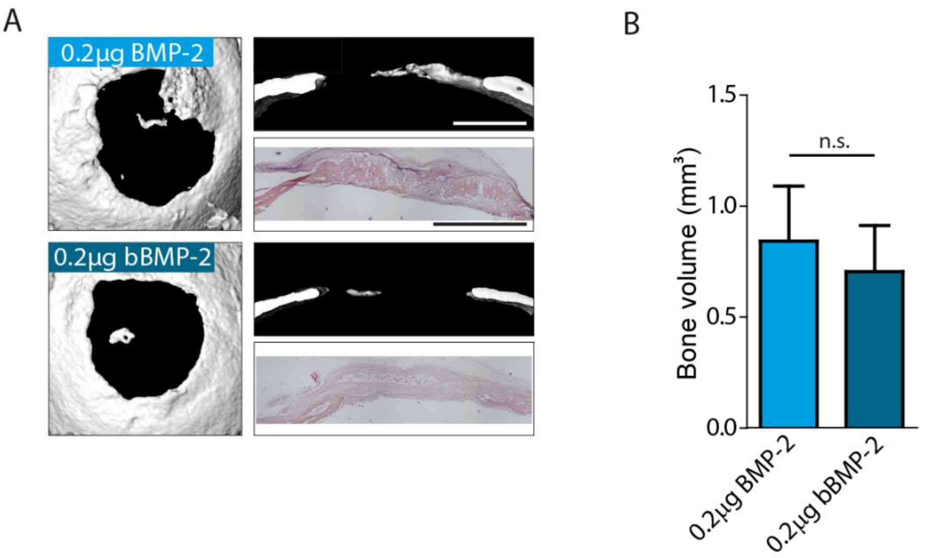

Figure S6. Osteoinductive effect of soluble versus bound BMP-2. Preformed hydrogel implants containing indicated amounts of soluble or matrix-immobilized BMP-2 were placed in ⌀ 4 mm bone defects created in the left and right parietal bones of C57BL/6 mice. (**A**) Representative top (left panels) and side views (upper right panels) of 3D surface rendered micro-CT measurements, as well as H&E stained coronal cross sections (lower right panel) using soluble BMP-2 or bound BMP-2 (scale bars = 1 mm). (**B**) Quantitative assessment of bone volume in response to different treatments. Data is depicted as mean ± SD for n = 6 independent defects. n.s. not significant (1-way ANOVA with Tukey-Kramer *post hoc* test).
